# High prevalence of kaolin consumption in migrant women living in a major urban area of France: A cross-sectional investigation

**DOI:** 10.1371/journal.pone.0220557

**Published:** 2019-07-31

**Authors:** Pascal Caillet, Maud Poirier, Marie Grall-Bronnec, Edouard Marchal, Alain Pineau, Catherine Pintas, Véronique Carton, Pascale Jolliet, Norbert Winer, Caroline Victorri-Vigneau

**Affiliations:** 1 Department of Clinical Pharmacology, University Hospital of Nantes, Nantes, France; 2 Department of Gynecology and Obstetrics, University Hospital of Nantes, Nantes, France; 3 Department of Addictology and Psychiatry, University Hospital of Nantes, Nantes, France; 4 SPHERE U1246 Unit, University of Nantes, University of Tours, INSERM, Nantes, France; 5 INRA, UMR 1280, Physiology of Nutritional Adaptations, University of Nantes, IMAD, Nantes, France; Universidad de Valladolid, SPAIN

## Abstract

Geophagia is a feeding behavior involving the regular intake of soil, including clay-like kaolin. Frequent in Africa, kaolin consumption is associated with heavy metal intoxication, iron and other micronutrient deficiencies, geohelminth infection and inactivation of concomitantly taken drugs. It is expected that this practice would be imported into an asylum country during the immigration process. To confirm this hypothesis, a single center, cross-sectional study was conducted at the University Hospital of Nantes, France, whose main objective was to assess whether the prevalence of kaolin consumers was high in a migrant population living in a large French metropolitan area (the city of Nantes). Each woman consulting for the first time at the Medical and Psychosocial Gynecology Obstetric Unit during the inclusion period ranging from January 1, 2017, to July 1, 2017, was asked for consent to be included in the study. The main outcome was the proportion of positive answers regarding consumption of kaolin within the last twelve months, with its 95% confidence interval (CI). A logistic regression was performed to identify drivers of consumption, and a clustering approach was conducted to identify profiles of consumers. A total of 284 women were included in the study, of whom 110 (38.7%) were pregnant. Our main finding was a 14.1% (95% CI: 10.5–18.6) prevalence of clay consumers. Second, the characteristic most strongly associated with consumption was Central or West Africa origin (adjusted Odds Ratio (aOR) = 52.7; 95% CI: 13.7–202.2). Finally, 60% of consumers showed signs of addictive-like phenomena, and three profiles were identified, depicting a continuum of patients in regard to their control over their kaolin consumption. Our results suggest that kaolin consumption is frequent in particular subpopulations of migrants. This warrants further study of the clinical consequences of kaolin consumption and its associated addictive-like symptoms.

## Introduction

Geophagia is a feeding behavior involving the regular intake of soil [1], including clay-like kaolin, an aluminum silicate often used in craft (e.g., pottery) or building material industry (e.g., brickyard). The prevalence of kaolin consumption in the general population of developed countries is poorly known. It seems to be frequently observed in people living in or originating from Africa [2] and specifically in populations such as pregnant women, children and people suffering from psychiatric disease [3]. The literature estimated its prevalence in pregnant African women to be between 14% [4] and 64% [5].

Different kaolin consumption habits have been documented, ranging from controlled intake in the context of traditional or alternative medicine practice to compulsive and uncontrollable intake occurring in particular physiological states such as pregnancy. Etiologies are still largely unknown. Regarding the specific case of consumption during pregnancy, one hypothesis is that it alleviates symptoms and discomfort associated with the early stages of pregnancy (hypersalivation, nausea, vomiting and pyrosis). Another hypothesis is that the occurrence of anemia triggers consumption, leading the patient to try to correct the deficiency by consuming other substances suspected to be rich in the element iron (although this iron may not be assimilable, as in the case with clay [6]). To date, none of these hypotheses has been validated [3].

Kaolin consumption is associated with various hazards. They include heavy metal intoxication (lead, arsenic and cadmium) [7], iron and other micronutrient deficiencies (zinc, selenium, copper) [8], geohelminth infection [9,10] and inactivation of concomitantly administered drugs [11]. A strong association has been found with anemia and increased risk of preterm birth and low birth weight [12]. Since this practice is largely unknown to French practitioners, it could delay the diagnosis and healing of consumers sick from their consumption (e.g., suffering from severe and chronic anemia) [13]. Moreover, some consumers seem to show addictive-like symptoms, whose magnitude and frequency are unknown. If addiction is confirmed, patients in this situation would need specific care to avoid relapse of consumption and reoccurrence of associated diseases [3].

### Objectives

The main objective of the current cross-sectional study was to quantify the prevalence of kaolin consumers in a primary care unit specialized in the handling of migrants in metropolitan France. Three other secondary objectives were investigated. First, we aimed to evaluate which factors were independently associated with kaolin consumption. Second, we evaluated the proportion of consumers showing potentially problematic consumption. Third, we searched for the existence of homogeneous profiles among the consumers and to describe any apparent profiles.

## Materials and methods

### Experimental design

A cross-sectional, single center study was conducted in the Medical and Psychosocial Gynecology Obstetric Unit of Nantes’ University Hospital (UGOMPS). The University Hospital of Nantes is a major health structure, providing care for most of the 3.5 million inhabitants of the French Pays-de-la Loire area. UGOMPS specializes in the care of vulnerable women, particularly those in the migration process. The inclusion period ranged from January 1, 2017, to July 1, 2017. This period was deemed sufficient to reach an adequate sample size.

### Participants and outcomes

Each woman consulting for the first time during the inclusion period was verbally asked for consent to be included in the study. In accordance with French laws and with the ethical committee that was consulted, collecting written consent was not necessary as the questionnaire was inserted into a medical consultation not specifically included for research purposes. In the case of consent, a face-to-face questionnaire was administered by the practitioner. An interpretation service was used if necessary (ISM Interprétariat, http://www.ism-interpretariat.fr/). Consent and results were recorded in the medical records of each patient. The questionnaire was designed to last less than 10 min because of feasibility constraints.

The primary outcome was the proportion of positive answers regarding the consumption of kaolin within the last twelve months, with its 95% confidence interval. The first secondary outcome was a set of factors identified as being associated with the consumption of kaolin within the last twelve months by use of a multivariate logistic regression, with their respective odds ratio and 95% confidence intervals. The second ancillary outcome regarding the evaluation of problematic consumption was the proportion of positive answers to items on a five-item kaolin problematic consumption screen. In the absence of dedicated and validated questionnaires regarding kaolin consumption that fit our feasibility constraints, questions were adapted from the CAGE questionnaire [14] designed for fast alcohol dependence screening with the help of an addictologist (Table 1).

**Table 1:** Questions used to investigate addictive-like symptomatology.

**Questions focusing the addictive-like symptomatology** - Have you ever had a desire to eat kaolin right away (Yes/No) ? - If so, how intense was the urge (between 1 to 10) ? - Have you ever had the impression of eating too much kaolin (Yes/No) ? - Have you ever wanted to lower or stop consuming kaolin (Yes/No) ? - Have you ever needed kaolin in the morning to feel fit (Yes/No) ? - Have you ever had any comments from people around you about eating kaolin (Yes/No) ?

A patient was declared to have a problematic consumption if she answered positively to at least two items among the five investigated (craving, feeling of excessive consumption, desire to stop or decrease, early morning need, negative reaction of relatives). The third ancillary outcome was a consumer profile and their description, identified by use of a clustering approach.

### Data collection

Clinical (age, BMI, history of and current disease, current pharmacotherapy, and pregnancy) and sociodemographic (geographical origin, living conditions, and social isolation) data were collected.

Living conditions were explored with a set of variables. We investigated whether the patient had children at charge, if she was isolated from her family (family isolation), in a relationship or socially inserted (e.g., meeting friends regularly). This variable was reduced to yes or no under the judgment of the physician. She was also asked if she had a stable address (stable housing). Her resources were evaluated by another set of questions, i.e., if she had a job or if she was a student, if she had all her care reimbursed (by being covered in the French healthcare system) and if she was a recipient of financial aid.

Kaolin consumption was evaluated in two steps. First, patients were asked whether they had ever consumed a substance displayed on a board filled with pictures of kaolin-based preparation and if she had consumed it within the last year. If the patient positively responded to the last question, other questions were asked: dates of the last episode of consumption, time of the day, frequency and volume of intake at its climax, whether it occurred during a pregnancy, how the substance was obtained, motivation to consume, if the patient triggered vomiting after consumption and if a replacement substance was used if kaolin was unavailable. The final questions were aimed specifically at the evaluation of a potential addictive symptomatology, as described above. The questionnaires in French and English are provided as supplementary materials, as well as the board with the pictures used to identify kaolin and approximate quantities that were consumed (supplementary files S1, S2 and S3 Figs respectively).

### Ethical issues

Ethical issues were checked by the independent local ethics committee (GNEDS for “Groupe Nantais d’Ethique dans le Domaine de la Santé”), which approved the conduct of this study and the verbal consent acquisition procedure. This study was also respectful of the French “Commission Nationale Informatique et Liberté” rules regarding data management and analysis.

### Statistical analysis

Characteristics of the population were described by the use of quantitative and qualitative variables. For normally distributed quantitative variables, the mean and standard deviation were used. For nonnormally distributed ranges, median and interquartile ranges were used. For qualitative variables, frequency and percentages were displayed. The primary analysis was the computation of the percentage of positive response to the question “Have you had consumed one of the substances displayed on this board within the last year?”, with its 95% exact confidence interval (95% CI), which was calculated according a binomial law (function binom.test, package “stats”, R software). A sample size of 139 patients was calculated to estimate this proportion with a reasonable precision, i.e., 10% ± 5%, according to data retrieved in the literature [15].

To evaluate drivers of kaolin consumption, we constructed a multivariable logistic regression model according to a backward stepwise approach. All variables showing a univariate association with a p-value < 0.75 were included in the initial model. Only variables showing a p-value ≤ 0.05 were kept in the final model. Adequate fit of the data and calibration were checked by performing a Hosmer and Lemeshow test and computing area under curve (AUC) and its 95% confidence interval. The handling of missing data was performed by conducting a complete case approach, as no variables involved in the analysis showed 10% or more missing values. The analysis was carried out with the “rms” R software package [16].

A cluster analysis was performed on the kaolin-consuming subgroup of our sample using a two-step approach [17,18]: first, a factorial analysis on mixed data (FAMD) [19] was performed, followed by a hierarchical ascending classification (HAC). The HAC was performed with factors constructed with the FAMD using Ward’s method and Euclidean distance [20]. The choice of the number of clusters for the HAC was done with the elbow rule. The analysis was performed with the “FactoMineR” R software package [21]. A descriptive analysis following the same template as described in the first paragraph was then performed, considering clusters as a grouping variable.

## Results and discussion

### Study participant description

A total of 284 women were included in the study. The characteristics of the sample are depicted in Table 2. Their median age was 30 years, and 39% (N = 110) were pregnant. Approximately two-thirds of the sampled women were in a relationship (N = 179, 63%) or with children at charge (N = 164, 58%) and approximately half of them were evaluated as socially inserted (N = 132, 46%). Approximately one-fifth of the sampled women were evaluated as being in a situation of family isolation (N = 48, 17%). Globally, a high level of precarity was observed, with only one-third having stable housing (N = 98, 35%) and one-half having a job (N = 127, 45%) or benefiting from support in resources (healthcare plan coverage for 39% and other financial support for 8%). The patients mostly originated in Africa (N = 163, 57%), especially from the West or Central Africa (WCA) area (N = 86, 31%, and N = 37, 13%, respectively). The characteristics of kaolin consumers and their consumption are shown in Table 3 and Table 4, respectively. Compared to nonconsumers, kaolin consumers were younger (median age = 26 years vs 31 years, p<0.01), more frequently pregnant (62.5% vs 34.8%, p<0.001) and originated in West Africa (60.0% vs 25.4%, p<0.001) and Central Africa (32.5% vs 9.8%, p<0.01). The consumers were less likely to have children at charge (40.0% vs 60.0%, p<0.01) and to be enrolled in a healthcare plan (17.5% vs 43%, p<0.001).

**Table 2:** Characteristics of the study population.

Variable	Category	Total number (N = 284)	Prevalence (95% confidence interval)	Median [IQR]	Missing (n, %)
Socioeconomic status	Children at charge	164	57.7 (51.9–63.3)		3 (1)
	Isolated from family	48	16.9 (13.0–21.7)		
	Socially inserted	132	46.5 (40.8–52.3)		
	In a relationship	179	63.0 (57.3–68.4)		
	Healthcare plan enrollee	112	39.4 (33.9–45.2)		
	Recipient of financial aid	22	7.7 (5.2–11.5)		
	Without a job	127	44.7 (39.0–50.5)		
	Student	16	5.6 (3.2–8.9)		
	Live in a stable housing	98	34.5 (29.2–40.2)		
Clinical variable	Age (year)			30 [25–38]	1 (0.3)
	BMI			25.3 [22.4–30.0]	16 (5.6)
	Pregnancy	110	38.7 (33.2–44.5)		3 (1)
Geographical origins	West Africa	86	31.2 (26.0–36.8)		8 (2.8)
	Europe	58	21.0 (16.6–26.2)		
	Central Africa	37	13.4 (9.9–17.9)		
	North Africa	25	9.1 (6.2–13.0)		
	Russia	23	8.3 (5.6–12.2)		
	Middle East	21	7.6 (5.0–11.3)		
	East Africa	15	5.4 (3.3–8.8)		
	Other	11	4.0 (2.2–7.0)		
Kaolin consumption	Ate kaolin at least once during lifetime	100	35.2 (29.9–40.9)		3 (1)
	Currently eat or ate kaolin in the past year	40	14.1 (10.5–18.6)		3 (1)

**Table 3:** Characteristics of the study population according to kaolin consumption.

Variable	Category	Non-consumers			Consumers			p-value*
		Total number (N = 244)	Prevalence (95% CI)	Median [IQR]	Total number (N = 40)	Prevalence (95% CI)	Median [IQR]	
Socioeconomic status	Children at charge	148	60.6 (54.2–66.8)		16	40.0 (24.9–56.7)		<0.01
	Isolated from her family	43	17.6 (13.0–23.0)		5	12.5 (4.0–26.8)		0.52
	Socially inserted	114	46.7 (40.3–53.2)		18	45.0 (29.2–61.5)		0.87
	In a relationship	155	63.5 (57.1–69.6)		24	60.0 (43.3–75.1)		0.73
	Healthcare plan enrollee	105	43.0 (36.7–49.5)		7	17.5 (7.3–32.8)		<0.001
	Recipient of financial aid	19	7.8 (4.7–11.9)		3	7.5 (1.6–20.4)		1.00
	Without a job	108	44.2 (37.9–50.7)		19	47.5 (31.5–63.9)		0.88
	Student	10	4.1 (2.0–7.4)		6	15 (5.7–29.8)		0.02
	Live in a stable housing	86	35.2 (29.2–41.6)		12	30.0 (16.6–46.5)		0.59
Clinical variable	Age (year)			31 [25–39]			26 [22–32]	<0.01
	BMI			25.4 [22.4–29.8]			24.5 [21.5–30.4]	0.42
	Pregnancy	85	34.8 (28.9–41.2)		25	62.5 (45.8–77.3)		<0.001
Geographical origins	Central Africa	24	9.8 (6.4–14.3)		13	32.5 (18.6–49.1)		<0.01
	East Africa	15	6.1 (3.5–9.9)		0	0 (0.0–0.9)		0.14
	North Africa	25	10.2 (6.7–14.7)		0	0 (0.0–0.9)		0.03
	West Africa	62	25.4 (20.1–31.3)		24	60.0 (43.3–75.1)		<0.001
	Europe	55	22.5 (17.4–28.3)		3	7.5 (1.6–20.4)		0.03
	Middle East	21	8.6 (5.4–12.8)		0	0 (0.0–0.9)		0.05
	Russia	23	9.4 (6.1–13.8)		0	0 (0.0–0.9)		0.05
	Other	11	4.5 (2.3–7.9)		0	0 (0.0–0.9)		0.23

This table depicts the results of the univariate analysis prior to conducting the multivariate analysis. All variables showing a p-value <0.75 were included in the initial step of the multivariate logistic regression.

* Pearson chi-square test for qualitative variables, Wilcoxon-Mann-Whitney test for quantitative variables

**Table 4:** Characteristics of kaolin consumers (N = 40) and their consumption.

Variable	Category	Total number	% (95% CI)
Consumption outside pregnancy	Yes	20	50.0 (35.2–64.8)
Sources	Friends	6	15.0 (7.1–29.1)
	Retailers	28	70.0 (54.6–81.9)
	Family	6	15.0 (7.1–29.1)
Approximated volume intake	<25 g	31	77.5 (62.5–87.7)
	25-<50 g	5	12.5 (5.5–26.1)
	50-<75 g	1	2.5 (0.4–12.9)
	75-<150 g	0	/
	≥150 g	1	2.5 (0.4–12.9)
Consumption frequency	Daily	9	22.5 (12.3–37.5)
	Weekly	14	35 (22.1–50.5)
	Monthly	5	12.5 (5.5–26.1)
	Occasionally	8	20 (10.5–34.8)
	Only once	3	7.5 (2.6–19.9)
Consumption motives	Cure	7	17.5 (8.7–31.9)
	Liking the smell	19	47.5 (32.9–62.5)
	Liking the taste	12	32.5 (20.1–48.0)
	Relaxation	8	20.0 (10.5–34.8)
	Baby protection	1	2.5 (0.4–12.9)
	Custom	8	20.0 (10.5–34.8)
	Other	12	32.5 (20.1–48.0)
Evaluation of the addictive symptomatology associated with the consumption	Craving	23	57.5 (42.2–71.5)
	Feeling of excessive consumption	13	32.5 (20.0–48.0)
	Want to stop or decrease consumption	23	57.5 (42.2–71.5)
	Need for consumption early in the morning	10	25.0 (14.2–40.2)
	Negative reactions of relatives	11	27.5 (16.1–42.8)
	At least 2 positive items among the 5 above	24	60.0 (44.6–73.6)

### Evaluation of the prevalence of consumers among the patients

Among the whole sample, 35% (95% CI: 29.9–40.9, N = 100) of the patients had eaten one of the forms of kaolin displayed on the board during their life, and 40 patients (14.1%, 95% CI: 10.5–18.6) were eating or had eaten kaolin within the past year.

### Motivations and modalities of consumption

Among pregnant consumers (N = 25/40), 80% reported consumption outside pregnancy (N = 20). Consumption mainly involved small quantities (approximately <25 g) consumed on a daily (N = 9, 22.5%) or weekly basis (i.e., every week, but not every day of the week, N = 14, 35.0%). The kaolin was obtained by buying from retailers (70%, N = 28), i.e., African or Asian good stores. The reported motives for consumption were mostly liking the smell or taste (respectively N = 19, 47.5% and N = 12, 32.5%), followed by relaxation purposes (N = 8, 20%), compliance to custom (N = 8, 20%) and seeking a cure (N = 7, 17.5%). Other motives were reported by 32.5% (N = 12) of the consumers and mainly included coping with nausea (N = 6) and decreasing salivation (N = 4).

### Evaluation of consumption-associated factors

Table 5 shows the results of the multivariable logistic regression. Adequacy and calibration criteria were satisfactory and allowed interpretation of the results (Hosmer-Lemeshow test result: p = 0.99; area under the receiver operator characteristic curve: 0.87, 95% CI(0.81–0.93)). Originating in WCA was significantly associated with consumption (adjusted OR (aOR) = 52.7, 95% CI(13.7–202.2)), suggesting a confounding relationship of WCA origin and kaolin consumption with other variables in the univariate analysis. An interaction was detected between having children at charge and being isolated from family. Having a dependent child was a protective factor against consumption only when the patient was not isolated from family (aOR = 0.12 (0.04–0.31)) and otherwise not related to consumption. On the other hand, being isolated from family was a protective factor only when the patient had no dependent children (aOR = 0.05 (0.01–0.21)), and not otherwise statistically related to consumption.

**Table 5:** Factors independently associated with kaolin consumption.

Variable	Category	Unadjusted OR (95% CI)	Adjusted OR (95% CI)	
Socioeconomic status	Children at charge	0.42 (0.21–0.82)	Effect of children at charge = Yes; when isolated from her family = Yes	1.92 (0.27–13.7)
			Effect of children at charge = Yes; when isolated from her family = No	0.12 (0.04–0.31)
	Isolated from her family	0.66 (0.24–1.78)	Effect of isolated from her family = Yes; with children at charge = Yes	0.90 (0.17–4.68)
			Effect of isolated from her family = Yes; with children at charge = No	0.05 (0.01–0.21)
	Socially inserted	0.91 (0.47–1.79)	/	
	In a relationship	0.83 (0.42–1.65)	/	
	Healthcare plan enrollee	0.27 (0.12–0.65)	/	
	Recipient of financial aid	0.95 (0.27–3.36)	/	
	Live in a stable housing	0.77 (0.37–1.60)	/	
	Student	4.08 (1.39–11.9)	/	
	Without a job	1.14 (0.59–2.23)	/	
Clinical variable	Age (year)	0.47 (0.27–0.81)	/	
	BMI	0.80 (0.49–1.29)	/	
	Pregnancy	3.11 (1.56–6.23)	/	
Geographical origins	Central or West Africa	21.5 (6.44–71.8)	52.7 (13.7–202.2)	

### Addictive-like symptoms

Among the 40 consumers, 24 patients (60%) answered positively at least 2 items among the 5 items (craving, feeling of excessive consumption, desire to stop or decrease, early morning need, negative reaction of relatives). Among them, 23 patients (57%) reported having had craving-like symptoms regarding kaolin consumption. When asked to score their craving intensity from 0 to 10, 17 (73.9%) scored over 5, and 7 (30.4%) scored 10. A feeling of excessive consumption was reported by 13 consumers (32.5%), and 23 (57.5%) reported a desire to stop or decrease consumption. Ten patients (25%) reported a need to consume early in the morning. A negative reaction of their relatives was reported by 11 consumers (27.5%).

### Identification of consumer profiles

The multivariate descriptive analysis of consumers (N = 40) identified three profiles in our sample (see supplementary figure S4 Fig for details of the elbow rule decision). Consumer characteristics are depicted in Table 6. The first cluster (N = 15) included women who were younger and more often pregnant but not with children at charge at the time of the consultation than the whole sample. They also reported addictive-like symptoms less often but more frequently a consumption motivated by custom. The second cluster (N = 20) included older consumers who were not pregnant but all with children at charge. A higher proportion of patients in this cluster compared to the first cluster reported addictive-like symptoms. The motives for consumption were liking the organoleptic properties of kaolin (smell and taste) and for relaxation purposes. The third cluster (N = 5) was a more severe variant of the second cluster, showing a more pronounced loss of control regarding their consumption.

**Table 6:** Description of clusters identified among kaolin consumers.

Variable	Category	Cluster 1 (N = 15)	Cluster 2 (N = 20)	Cluster 3 (N = 5)	p-value*	p-trend**
Need to consume early in the morning (N, %)	Yes	5 (33.3)	0 (0.0)	5 (100.0)	0.0005	0.017
Pregnancy (N, %)	Yes	14 (93.3)	11 (55.0)	0 (0.0)	0.001	0.0001
Consumption motivated by liking of smell (N, %)	Yes	2 (13.3)	12 (60.0)	5 (100.0)	0.001	0.0002
Children at charge (N, %)	Yes	1 (7.0)	10 (50.0)	5(100.0)	0.0015	0.0014
Feeling of excessive consumption (N, %)	Yes	3 (20.0)	5 (25.0)	5 (100.0)	0.0015	0.001
Want to stop or decrease consumption (N, %)	Yes	7 (46.7)	19 (95.0)	5 (100.0)	0.002	0.007
Consumption motivated by need for relaxation (N, %)	Yes	1 (0.7)	3 (15.0)	4 (80.0)	0.003	0.001
Consumption motivated by liking of taste (N, %)	Yes	0 (0.0)	10 (50.0)	3 (60.0)	0.003	0.003
Consumption motivated by custom (N, %)	Yes	7 (46.8)	0 (0.0)	1 (20.0)	0.005	0.001
Consumption motivated by another motive (N, %)	Yes	9 (60.0)	3 (15.0)	0 (0.0)	0.0065	0.017
Age (median, IQR)	/	22 [18.5–26]	29 [23–33]	32 [25–37]	0.007	0.002
Negative reactions of relatives (N, %)	Yes	1 (6.7)	6 (30.0)	4 (80.0)	0.0075	0.002
Recipient of financial aid (N, %)	Yes	2 (13.3)	2 (10.0)	3 (60.0)	0.049	NS
Isolated from her family (N, %)	Yes	0 (0.0)	5 (25.0)	0 (0.0)	0.08	NS
Craving (N, %)	Yes	9 (60.0)	17 (85.0)	5 (100.0)	0.1	NS
Consumption as a cure (N, %)	Yes	3 (20.0)	3 (15.0)	1 (20.0)	0.17	NS
Without a job (N, %)	Yes	5 (33.3)	12 (60.0)	2 (40.0)	0.28	NS
Live in a stable housing (N, %)	Yes	4 (26.7)	5 (25.0)	3 (60.0)	0.38	NS
Consumption motivated by baby protection (N, %)	Yes	1 (6.7%)	0 (0.0)	0 (0.0)	0.5	NS
Socially inserted (N, %)	Yes	7 (46.7)	10 (50.0)	1 (20.0)	0.61	NS
West or Central Africa origin (N, %)	Yes	13 (86.7)	19 (95.0)	5 (100.0)	0.72	NS
In a relationship (N, %)	Yes	10 (66.7)	11 (55.0)	3 (60.0)	0.9	NS

* Pearson chi-square test for qualitative variables and Kruskal-Wallis test for quantitative variables; statistical significance threshold with Hochberg correction for multiple tests

** Cochran-Armitage test for linear trends for qualitative variables and Jonckheere trend test for quantitative variables

Our main finding was that 14.1% of patients consulting in a major French metropolitan urban area care center specialized in the care of psychologically and socially frail migrant women were clay consumers. Second, the characteristic most strongly associated with consumption was Central or West Africa origin (aOR = 52.7; 95% CI (13.7–202.2)), confirming the strong cultural component of the practice. Finally, a high proportion of consumers (60%) showed addictive-like symptoms, and three profiles were identified, depicting a continuum of patients in regard to their control over kaolin consumption.

The prevalence of geophagia is already known to be high in sub-Saharian Africa. According to different studies, the prevalence could range from 14% to more than 60% in the population living in this area [4,5]. The prevalence in European countries is thought to be far lower. In a Danish cohort study including 100 000 pregnant women conducted in 2006, the prevalence of pica was evaluated to be 0.02% [22]. Our study confirmed that this practice is associated with a strong cultural component [23] and may be far more frequent in specific subgroups of populations. Moreover, our results suggest that this consumption is often exported to foreign countries when migration occurs and may become concentrated in precarious and less visible but highly vulnerable populations in asylum countries, turning this into a stealthy and underestimated practice.

Practitioners in asylum countries are largely unaware of their patient’s consumption; this problem becomes more pronounced as the patients themselves and their relatives have a negative view making this an habit hard to unveil and break [24]. This poor knowledge regarding geophagia is prone to have consequences following a delay in identification and treatment in practice. The consequences are various and include deficiencies by chemical chelation of nutrients (Fe, zinc, copper, and selenium), heavy metal intoxication (lead, cadmium and arsenic), geohelminth infection, gastrointestinal upset, dental injury and achlorhydria [10,25–27]. Whether consuming during pregnancy can affect both mother and child remains debatable [28–31], but some evidence has indicated potential lead poisoning of fetuses [32]. The lead content of kaolin is highly variable, but a sample bought in a retail shop frequently mentioned by patients showed a lead concentration of 36 ppm, which is worrisome. Moreover, as clay could chelate chemical components that are concomitantly consumed and inhibit their absorption, it could also impair the function numerous oral pharmacotherapies [33,13], e.g., oral ferrous supplementation prescribed to correct or prevent an iron deficiency anemia, which may have been induced by kaolin consumption. A solution for preventing clay consumption could be enhancing the nutritional knowledge of consumers. In a pilot study that included 135 geophagous women in rural Nigeria, nutrition education focusing on geophagia significantly decreased the practice in 77% of the participants [34].

However, the presence of addictive-like symptoms seems frequent and could impair the outcomes of such interventions. In our study, we found that 60% of recent consumers responded positively to at least 2 items regarding potential addictive-like behavior, with 23 (57%) reporting a craving. In a qualitative study conducted in a sample of 35 geophagous patients, 25 (71%) attributed their consumption to the occurrence of craving [23]. Withdrawal symptoms such as increased heartbeat, restlessness, dysphoria, dissatisfaction, hypersalivation and nervousness were also reported. In both studies, smell was identified as a trigger of cravings.

Classifying kaolin consumption as a pica is tentative. According to the DSM-5, a pica is essentially defined by the ingestion of a non-nutritive or nonedible substance that persists for at least one month in an individual aged over 2 years old [1]. Other criteria include the fact that this behavior is not adequate to the developmental level of the patient and that this behavior is not a culturally or socially acceptable practice. Regarding kaolin consumption, the cultural acceptance of consumption is debatable as it seems to be perceived with ambivalence within the practicing communities. Clay consumption is associated with low education by men and appears to be a socially sanctioned practice. On the other hand, there appears to be significant social pressure to desire clay during pregnancy in certain cultures [24]. Finally, some data suggest that it preferentially occurs during pregnancy and then naturally vanishes thereafter [3], without a withdrawal syndrome; this latter finding questions the definition of kaolin consumption as a true addiction.

From a pharmacological perspective, the composition of clay is not suggestive of a psychotropic effect of the substance. Kaolinite is mainly composed of silicon, aluminum and iron. Silicon is not known to possess psychotropic activity. A debate exists regarding silicon toxicity in the brain when administered in nanoparticles, but these are not naturally present in clay [35,36]. Aluminum is known to be neurotoxic at very high serum concentrations (e.g., causing encephalopathy in patients performing dialysis on a regular basis), and its association with neurodegenerative diseases has been debated [37]. However, no information is available regarding activation of reward circuitry during aluminum intoxication.

The fact that iron deficiency anemia can facilitate the development of addictive symptomatology by creating or reinforcing iron deficiency anemia has to be discussed. Evidence favoring this hypothesis is that the intake of clay hinders the absorption of iron and L-tyrosine is transformed into L-DOPA by the enzyme tyrosine hydroxylase, which requires iron as a cofactor [38]. Therefore, iron deficiency anemia is theoretically likely to disrupt the dopaminergic system in the brain [39–42]. Many structures are thought to be potentially affected, including mesocortical, mesolimbic, nigrostriatal and tuberohypophyseal pathways [43]. Thus, anemia could be linked to the development of addictive symptomatology through a mechanism whose detail is still unknown but potentially mediated by dysregulation of dopamine transmission in reward and prefrontal circuits [44]. An interesting clinical element in this setting is the observation in the literature of patients showing cravings for various non-nutritive substances that is triggered by olfactory stimuli, which disappears when the iron deficiency is corrected [45,46], as well as at least one reported case of craving regarding kaolin consumption [13].

Our cluster analysis provided further information. The identified clusters depicted a continuum of control regarding consumption. The patients in the first cluster may correspond to younger women initiating their consumption due to discomfort induced by pregnancy and their beliefs in the therapeutic properties of clay (reward-driven consumption). The patients in the second and third clusters are more advanced in their life and continue to consume kaolin without the need to relieve discomfort associated with pregnancy. These women may correspond to patients more advanced in their problematic use of clay and present a more habit-driven consumption than the patients belonging to the first cluster. We also observed that clusters 2 and 3 less often reported a consumption motivated by custom compared to cluster 1, which was composed of younger women. This poses the question of a potential effect of acculturation in French society regarding changes in the perception of this practice.

Some recommendations can be discussed regarding the handling of geophagous patients, as explained in the paper of Lambert et al [3]. Questioning pica must be systematic during antenatal consultation, all the more if there is anemia, a psychiatric disease or belonging to an ethnic minority. Modifiable factors associated with consumption must be investigated and corrected (dyspepsia, nausea, iron deficiency anemia and stress). Mechanical complications (e.g., constipation) must be investigated and treated, e.g., with lubricants. Potassium levels must be checked, as well as heavy metal intoxication (at least lead to rule out lead poisoning). If an iron deficiency anemia is diagnosed, the efficacy of oral supplementation must be checked, and in cases of inefficacy, intravenous iron supplementation or maternal transfusion must be considered. Regarding alimentation or drug administration, they must be shifted (at least 2 hours) from the intake of clay if consumption could not be stopped. In the case of addictive symptomatology, seeking the support of an addiction team can be beneficial, and as a last resort, switching towards another type of pica (starch, ice) could be advised to avoid the toxicological consequences of clay consumption.

A main limitation of our study is that, despite being interesting, the data regarding the addictive-like symptomatology are only partly documented. One reason is that documentation of addictive-like phenomena was a secondary objective of our study. A second reason is that our methodology involved questioning patients during a clinical consultation that was not dedicated to the study of addiction-like phenomena. Therefore, time constraints were very tight, as only a few minutes of the whole clinical interview were dedicated to the relevant questionnaire. This process did not allow a complete evaluation of the addictive-like symptomatology but rather a screening. However, although the addictive-like symptomatology evaluation was incomplete, our results warrant further studies dedicated to the full evaluation of this addictive-like symptomatology, and if the presence of addiction is confirmed, its accurate management could be a major step towards preventing the consequences of kaolin consumption in migrant populations.

There are other limitations to our study. First, although we carefully chose potential covariates to be included prior to the analysis, there is always the possibility of the presence of residual confounding, which could at least partially explain the observed associations. Second, we did not record patients who refused to participate in our study, which could have biased our results and restricted the generalizability of our study (in most instances, patients who were not questioned were in a critical situation rendering such questioning inappropriate). Last, our work could have missed some consumers due to that the board displaying the pictures of kaolin was not exhaustive.

## Conclusions

This study documented the prevalence of kaolin consumption in a population of migrants. It showed that in this population, the consumption of kaolin occurs in a non-negligible proportion of patients. This knowledge is important because migratory flux towards Europe increases in magnitude, and Western caregivers are often poorly aware of this practice, which may impair care. This finding warrants further study of the addictive symptoms often associated with the consumption of kaolin.

## Supporting information

S1 Fig French questionnaire. This is the original questionnaire used in the study.
(PDF)

S2 Fig English questionnaire. This is an English translation of the original questionnaire used in the study.
(PDF)

S3 Fig Picture board. This is the picture board used to identify kaolin consumption and evaluate quantities consumed.
(PDF)

S4 Fig Choice of clusters using the elbow rule. This figure shows how the 3-cluster solution was chosen.
(PDF)

## References

[1] Association AP. Diagnostic and Statistical Manual of Mental Disorders, 5th Edition: DSM-5. Washington, D.C: American Psychiatric Publishing; 2013.

[2] Young SL. Craving Earth: Understanding Pica—the Urge to Eat Clay, Starch, Ice, and Chalk. Columbia University Press; 2011.

[3] Lambert V, Pouget K, Basurko C, Boukhari R, Dallah F, Carles G. [Geophagy and pregnancy: current knowledge and management. Clinical experiences of an obstetrical department in French Guiana]. J Gynecol Obstet Biol Reprod (Paris). 2014;43: 496–503. 10.1016/j.jgyn.2013.06.001 23871612

[4] Osman AK. Dietary practices and aversions during pregnancy and lactation among Sudanese women. J Trop Pediatr. 1985;31: 16–20. 10.1093/tropej/31.1.16 3981689

[5] Nyaruhucha CNM. Food cravings, aversions and pica among pregnant women in Dar es Salaam, Tanzania. Tanzan J Health Res. 2009;11: 29–34. 19445102

[6] Seim GL, Ahn CI, Bodis MS, Luwedde F, Miller DD, Hillier S, et al Bioavailability of iron in geophagic earths and clay minerals, and their effect on dietary iron absorption using an in vitro digestion/Caco-2 cell model. Food Funct. 2013;4: 1263–1270. 10.1039/c3fo30380b 23787405 PMC4033299

[7] Lar UA, Agene JI, Umar AI. Geophagic clay materials from Nigeria: a potential source of heavy metals and human health implications in mostly women and children who practice it. Environ Geochem Health. 2015;37: 363–375. 10.1007/s10653-014-9653-0 25416852

[8] Hooda PS, Henry CJK, Seyoum TA, Armstrong LDM, Fowler MB. The potential impact of soil ingestion on human mineral nutrition. Sci Total Environ. 2004;333: 75–87. 10.1016/j.scitotenv.2004.04.023 15364520

[9] Kutalek R, Wewalka G, Gundacker C, Auer H, Wilson J, Haluza D, et al Geophagy and potential health implications: geohelminths, microbes and heavy metals. Trans R Soc Trop Med Hyg. 2010;104: 787–795. 10.1016/j.trstmh.2010.09.002 20889178

[10] Odongo AO, Moturi WN, Mbuthia EK. Heavy metals and parasitic geohelminths toxicity among geophagous pregnant women: a case study of Nakuru Municipality, Kenya. Environ Geochem Health. 2016;38: 123–131. 10.1007/s10653-015-9690-3 25750054

[11] Awad ME, López-Galindo A, Setti M, El-Rahmany MM, Iborra CV. Kaolinite in pharmaceutics and biomedicine. Int J Pharm. 2017;533: 34–48. 10.1016/j.ijpharm.2017.09.056 28943206

[12] Allen LH. Anemia and iron deficiency: effects on pregnancy outcome. Am J Clin Nutr. 2000;71: 1280S–4S. 10.1093/ajcn/71.5.1280s 10799402

[13] Pain S, Fauconneau B, Bouquet E, Vasse-Terrier L, Pérault-Pochat M-C. Severe craving associated with kaolin consumption. Eat Weight Disord. 2018; 10.1007/s40519-018-0583-1 30255289

[14] Ewing JA. Detecting alcoholism. The CAGE questionnaire. JAMA. 1984;252: 1905–1907. 10.1001/jama.252.14.1905 6471323

[15] Kmiec I, Nguyen Y, Rouger C, Berger JL, Lambert D, Hentzien M, et al Factors Associated with Geophagy and Knowledge About Its Harmful Effects Among Native Sub-Saharan African, Caribbean and French Guiana HIV Patients Living in Northern France. AIDS Behav. 2016; 10.1007/s10461-016-1661-x 28028652

[16] Helmreich JE. Regression Modeling Strategies with Applications to Linear Models, Logistic and Ordinal Regression and Survival Analysis (2nd Edition). Journal of Statistical Software. 2016;70 10.18637/jss.v070.i08

[17] Ball GH, Hall DJ. A clustering technique for summarizing multivariate data. Behav Sci. 1967;12: 153–155. 6030099 10.1002/bs.3830120210

[18] McLachlan GJ. Cluster analysis and related techniques in medical research. Stat Methods Med Res. 1992;1: 27–48. 10.1177/096228029200100103 1341650

[19] Pagès J. [Factorial analysis on mixed data]. Revue Statistique Appliquee. 2004;52: 93–111.

[20] Ward JH. Hierarchical Grouping to Optimize an Objective Function. Journal of the American Statistical Association. 1963;58: 236 10.2307/2282967

[21] Lê S, Josse J, Husson F. FactoMineR: An R Package for Multivariate Analysis. Journal of Statistical Software. 2008;25 10.18637/jss.v025.i01

[22] Mikkelsen TB, Andersen A-MN, Olsen SF. Pica in pregnancy in a privileged population: myth or reality. Acta Obstet Gynecol Scand. 2006;85: 1265–1266. 10.1080/00016340600676425 17068688

[23] Huebl L, Leick S, Guettl L, Akello G, Kutalek R. Geophagy in Northern Uganda: Perspectives from Consumers and Clinicians. Am J Trop Med Hyg. 2016;95: 1440–1449. 10.4269/ajtmh.15-0579 27698274 PMC5154465

[24] Hunter-Adams J. Interpreting habits in a new place: Migrants’ descriptions of geophagia during pregnancy. Appetite. 2016;105: 557–561. 10.1016/j.appet.2016.06.033 27364379

[25] Wilson RL, Grieger JA, Bianco-Miotto T, Roberts CT. Association between Maternal Zinc Status, Dietary Zinc Intake and Pregnancy Complications: A Systematic Review. Nutrients. 2016;8 10.3390/nu8100641 27754451 PMC5084028

[26] Njiru H, Elchalal U, Paltiel O. Geophagy during pregnancy in Africa: a literature review. Obstet Gynecol Surv. 2011;66: 452–459. 10.1097/OGX.0b013e318232a034 21944157

[27] Al-Rmalli SW, Jenkins RO, Watts MJ, Haris PI. Risk of human exposure to arsenic and other toxic elements from geophagy: trace element analysis of baked clay using inductively coupled plasma mass spectrometry. Environ Health. 2010;9: 79 10.1186/1476-069X-9-79 21182763 PMC3022881

[28] Corbett RW, Ryan C, Weinrich SP. Pica in pregnancy: does it affect pregnancy outcomes? MCN Am J Matern Child Nurs. 2003;28: 183–189; quiz 190–191. 12771697 10.1097/00005721-200305000-00009

[29] Levy A, Fraser D, Katz M, Mazor M, Sheiner E. Maternal anemia during pregnancy is an independent risk factor for low birthweight and preterm delivery. Eur J Obstet Gynecol Reprod Biol. 2005;122: 182–186. 10.1016/j.ejogrb.2005.02.015 16219519

[30] Kavle JA, Stoltzfus RJ, Witter F, Tielsch JM, Khalfan SS, Caulfield LE. Association between anaemia during pregnancy and blood loss at and after delivery among women with vaginal births in Pemba Island, Zanzibar, Tanzania. J Health Popul Nutr. 2008;26: 232–240. 18686556 PMC2740668

[31] Hamilton S, Rothenberg SJ, Khan FA, Manalo M, Norris KC. Neonatal lead poisoning from maternal pica behavior during pregnancy. J Natl Med Assoc. 2001;93: 317–319. 11560285 PMC2593967

[32] Gundacker C, Kutalek R, Glaunach R, Deweis C, Hengstschläger M, Prinz A. Geophagy during pregnancy: Is there a health risk for infants? Environ Res. 2017;156: 145–147. 10.1016/j.envres.2017.03.028 28342960

[33] von Garnier C, Stünitz H, Decker M, Battegay E, Zeller A. Pica and refractory iron deficiency anaemia: a case report. J Med Case Rep. 2008;2: 324 10.1186/1752-1947-2-324 18838005 PMC2567333

[34] Iron-Segev S, Lusweti JN, Kamau-Mbuthia E, Stark AH. Impact of Community-Based Nutrition Education on Geophagic Behavior and Dietary Knowledge and Practices among Rural Women in Nakuru Town, Kenya: A Pilot Study. J Nutr Educ Behav. 2017; 10.1016/j.jneb.2017.10.013 29277491

[35] Salem A, Oudhabechi A, Sakly M. Effect of Nano-sized SiO2 particles in Rats: Cognitive function and biochemical response. Arch Environ Occup Health. 2018; 1–19. 10.1080/19338244.2018.1489365 29920170

[36] Zulfiqar U, Subhani T, Husain SW. Synthesis and characterization of silica nanoparticles from clay. Journal of Asian Ceramic Societies. 2016;4: 91–96. 10.1016/j.jascer.2015.12.001

[37] Inan-Eroglu E, Ayaz A. Is aluminum exposure a risk factor for neurological disorders? J Res Med Sci. 2018;23: 51 10.4103/jrms.JRMS_921_17 30057635 PMC6040147

[38] Bianco LE, Wiesinger J, Earley CJ, Jones BC, Beard JL. Iron deficiency alters dopamine uptake and response to L-DOPA injection in Sprague-Dawley rats. J Neurochem. 2008;106: 205–215. 10.1111/j.1471-4159.2008.05358.x 18363828

[39] Pino JMV, da Luz MHM, Antunes HKM, Giampá SQ de C, Martins VR, Lee KS. Iron-Restricted Diet Affects Brain Ferritin Levels, Dopamine Metabolism and Cellular Prion Protein in a Region-Specific Manner. Front Mol Neurosci. 2017;10: 145 10.3389/fnmol.2017.00145 28567002 PMC5434142

[40] Matak P, Matak A, Moustafa S, Aryal DK, Benner EJ, Wetsel W, et al Disrupted iron homeostasis causes dopaminergic neurodegeneration in mice. Proc Natl Acad Sci USA. 2016;113: 3428–3435. 10.1073/pnas.1519473113 26929359 PMC4822577

[41] Unger EL, Bianco LE, Jones BC, Allen RP, Earley CJ. Low brain iron effects and reversibility on striatal dopamine dynamics. Experimental Neurology. 2014;261: 462–468. 10.1016/j.expneurol.2014.06.023 24999026 PMC4318655

[42] Youdim MBH. Brain iron deficiency and excess; cognitive impairment and neurodegeneration with involvement of striatum and hippocampus. Neurotox Res. 2008;14: 45–56. 18790724 10.1007/BF03033574

[43] Lozoff B. Early iron deficiency has brain and behavior effects consistent with dopaminergic dysfunction. J Nutr. 2011;141: 740S–746S. 10.3945/jn.110.131169 21346104 PMC3056585

[44] Smith RJ, Laiks LS. Behavioral and neural mechanisms underlying habitual and compulsive drug seeking. Progress in Neuro-Psychopharmacology and Biological Psychiatry. 2018;87: 11–21. 10.1016/j.pnpbp.2017.09.003 28887182 PMC5837910

[45] Hansen BR, Bottner WA, Ravindran A, DeJesus R, Go RS. Desiderosmia (olfactory craving): A novel symptom associated with iron deficiency anemia. Am J Hematol. 2017;92: E93–E94. 10.1002/ajh.24706 28240803

[46] Hansen BR, Bottner WA, Ravindran A, DeJesus R, Go RS. A follow-up on desiderosmia (olfactory craving), a novel symptom associated with iron deficiency anemia. Am J Hematol. 2017;92: E546 10.1002/ajh.24806 28240803

