# Supplementary material for: High prevalence of kaolin consumption in migrant women living in a major urban area of France: A cross-sectional investigation
Source: PLoS One. 2019 Jul 31;14(7):e0220557. doi: 10.1371/journal.pone.0220557 (PMC6668907; doi:10.1371/journal.pone.0220557)
Supplement: S1 Fig — This is the original questionnaire used in the study. (PDF) [file pone.0220557.s001.pdf]

RENSEIGNEMENTS DEMOGRAPHIQUES

1. **Âge en année**

2. **Situation familiale de la patiente** (*Plusieurs réponses possibles.)*

   ☐ Entourée ☐ Isolée socialement ☐ Enfants à charge ☐ En couple

3. **Condition de vie** (*Plusieurs réponses possibles.)*

   ☐ En emploi
   ☐ Etudiante
   ☐ Sans emploi, mais compagnon en emploi
   ☐ Sans emploi, compagnon sans emploi
   ☐ Allocataire d'aides : ……………………
   ☐ Bénéficiaire d'une mutuelle
   ☐ Habite un logement de façon stable
   ☐ Autre :......................

4. **Origine géographique de la patiente (pays) :**

5. **Origine géographique des parents de la patiente :**

   Pays du père :

   Pays de la mère :

RENSEIGNEMENTS CLINIQUES

6. **Poids en kg (ex: 62) :**
7. **Taille en cm (ex: 172) :**
8. **Date dernière règles :**
9. **Si suivi obstétrique, date de début de grossesse retenue :**
10. **Traitements en cours - Médicaments et/ou substances associés**

11. **Problèmes de santé existants**

CONSOMMATION D'ARGILE

12. **Avez-vous mangé une des substances sur les images au cours de votre vie ?**

☐Oui ☐Non

13. **Si oui, comment vous l'appeliez ?**

14. **Si oui, à quel âge en avez-vous mangé pour la première fois ?**

15. **Si oui, à quand date la dernière prise ?**

16. **Sous quelle forme vous le mangiez? (***Plusieurs réponses possibles)*

☐ Pierre ☐ Poudre ☐ Pâte ☐ Autre

17. **Comment vous le prépariez ?**

18. **Avez-vous mangé une des substances sur les images durant l'année écoulée ?**

☐Oui ☐Non

SI REPONSE NON A LA QUESTION PRECEDENTE, NE PAS REMPLIR LA SUITE DU QUESTIONNAIRE

19. **Si oui, quelle est la date de début et éventuellement de fin de la dernière période où vous en avez mangé ?**

Date de début : Date de fin :

20. **Au cours de la dernière période de consommation, au maximum de cette consommation, à quelle fréquence le mangiez-vous ? (***Une seule réponse possible)*

☐ Tous les jours, voire plusieurs fois par jour
☐ Pas tous les jours, mais plusieurs fois par semaine
☐ Pas toutes semaines, mais plusieurs fois par mois
☐ Occasionnellement (une fois par mois, voire moins)
☐ Une seule fois

21. **Si vous êtes enceinte, est-ce que vous en mangez en dehors des moments où vous êtes enceinte ?**

☐ Non-applicable ☐ Oui ☐ Non

22. **A quel moment de la journée le mangez-vous ?**

23. **Comment est-ce que vous l'obtenez ?**

☐ Amis

☐ Famille

☐ Commerçant. Si oui lequel : ……………………….

☐ Autre :

24. **Quel volume vous en mangez dans une journée ? (***Une seule réponse possible)*

☐ 1 ☐ 2 ☐ 3 ☐ 4 ☐ 5

25. **Pour quelles raisons en avez-vous mangé ou en mangez-vous ?**

☐ Habitude ou coutume dans ma famille
☐ C'est un remède à : …………………..
☐ Ça protège mon bébé des maladies
☐ Ça me détend
☐ J'aime l'odeur
☐ J'aime le goût
☐ C'est l'occasion de partager avec mes proches
☐ Autre :

26. **Est-ce que parfois vous vous faites vomir après en avoir mangé ?**

☐Oui ☐Non

27. **Si vous voulez en manger tout de suite mais vous n'en avez pas sur vous, est-ce que vous mangez quelque chose d'autre à la place ?**

☐Oui ☐Non

28. **Si oui, quoi ?**

Evaluation craving - dépendance lors du dernier épisode de conso

29. **Est-ce qu'il vous est déjà arrivé dans la journée d'avoir une envie de manger tout de suite du Kaolin ?**

☐Oui ☐Non

30. **Si oui, quelle était l'intensité de cette envie ?**
Cocher la case la plus adaptée pour représenter l'intensité de votre envie.
Une seule réponse possible.

	1	2	3	4	5	6	7	8	9	10	
**Envie faible et très facilement gérable**											**Envie très forte et incontrôlable**

31. **Est-ce que vous avez déjà eu l'impression de manger trop de kaolin ?**

☐Oui ☐Non

32. **Est-ce que vous avez déjà eu envie d'abaisser ou arrêter votre consommation de Kaolin ?**

☐Oui ☐Non

33. **Est-ce que vous avez déjà eu besoin de Kaolin dès le matin pour vous sentir en forme ?**

☐Oui ☐Non

34. **Votre entourage vous a-t-il déjà fait des remarques sur le fait que vous mangiez du Kaolin?**

☐Oui ☐Non

Dosages biologiques prescrits :
