# Supplementary material for: High prevalence of kaolin consumption in migrant women living in a major urban area of France: A cross-sectional investigation
Source: PLoS One. 2019 Jul 31;14(7):e0220557. doi: 10.1371/journal.pone.0220557 (PMC6668907; doi:10.1371/journal.pone.0220557)
Supplement: S2 Fig — This is an English translation of the original questionnaire used in the study. (PDF) [file pone.0220557.s002.pdf]

DEMOGRAPHIC DATA

1. **Age in years**

2. **Patient's family situation** *(Several possible answers)*

☐ Surrounded ☐ Socially isolated ☐ Dependent children ☐ In a couple

3. **Living conditions** *(Several possible answers)*

☐ Employed
☐ Student
☐ Unemployed, partner is employed
☐ Unemployed, partner is unemployed
☐ Aid recipient: ……………………
☐ Beneficiary of a mutual insurance company
☐ Lives in a dwelling in a stable way
☐ Other:......................

4. **Geographical origin of the patient (country):**

5. **Geographical origin of the patient's parents:**

Father's country:

Mother's country:

CLINICAL DATA

6. **Weight in kg (ex: 62):**

7. **Height in cm (ex: 172):**

8. **Date of last period:**

9. **If obstetrical follow-up, the determined date of onset of pregnancy:**

10. **Ongoing treatments - Drugs and/or related substances**

11. **Existing health problems**

KAOLIN CONSUMPTION

12. **Have you eaten any of the substances in the images in your life?**

☐Yes ☐No

13. **If so, what did you call it?**

14. **If so, at what age did you first eat it?**

15. **If so, when was the date of last intake?**

16. **In what form did you eat it?** *(Several possible answers)*

☐ Stone ☐ Powder ☐ Paste ☐ Other

17. **How did you prepare it?**

18. **Have you eaten any of the substances in the images in the past year?**

☐Yes ☐No

IF NO TO THE PREVIOUS QUESTION, DO NOT COMPLETE THE REST OF THE QUESTIONNAIRE

19. **If so, what was the start and possibly end date of the last period you ate it?**

Start date: End date:

During the last consumption period, when most of this consumption occurred, how often did you eat it? (*Only one answer)*

☐ Every day, or even several times a day
☐ Not every day, but several times a week
☐ Not every week, but several times a month
☐ Occasionally (once a month or less)
☐ Only once

20. **If you are pregnant, do you eat it when you were not pregnant?**

☐ Not applicable ☐ Yes ☐ No

21. **At what time of day do you eat it?**

22. **How do you get it?** *(Several possible answers)*

☐ Friends

☐ Family

☐ Trader. If so, which one: ……………………….

☐ Other:

23. **How much do you eat in a day? (***Only one answer)*

☐ 1 ☐ 2 ☐ 3 ☐ 4 ☐ 5

24. **Why did you eat it or do you eat it?**

☐ Habits or customs in my family
☐ It's a cure for: …………………..
☐ It protects my baby from diseases
☐ It relaxes me
☐ I like the smell
☐ I like the taste
☐ It's an opportunity to share with my family and friends
☐ Other:

25. **Do you sometimes make yourself vomit after eating it?**

☐Yes ☐No

26. **If you want to eat it right away but you don't have it on you, do you eat something else instead?**

☐Yes ☐No

27. **If so, what ?**

Craving - addiction evaluation during the last episode of consumption

28. **Have you ever had a desire to eat kaolin right away?**

☐Yes ☐No

29. **If so, how intense was the urge?**
Check the box that is most appropriate to represent the intensity of the desire.
Only one possible answer.

	1	2	3	4	5	6	7	8	9	10	
**Low desire and very easily manageable**											**Very strong and uncontrollable desire**

30. **Have you ever had the impression of eating too much kaolin?**

☐Yes ☐No

31. **Have you ever wanted to lower your consumption or stop consuming kaolin?**

☐Yes ☐No

32. **Have you ever needed kaolin in the morning to feel fit?**

☐Yes ☐No

33. **Have you ever had any comments from people around you about eating kaolin?**

☐Yes ☐No

Prescribed biological dosages:
