# Supplementary figures and images for: High prevalence of kaolin consumption in migrant women living in a major urban area of France: A cross-sectional investigation
Source: PLoS One. 2019 Jul 31;14(7):e0220557. doi: 10.1371/journal.pone.0220557 (PMC6668907; doi:10.1371/journal.pone.0220557)

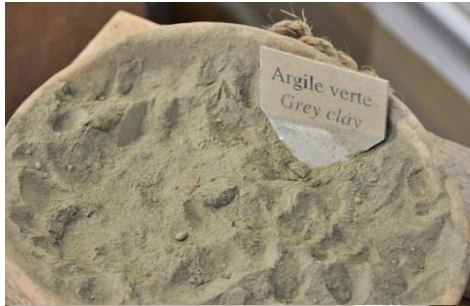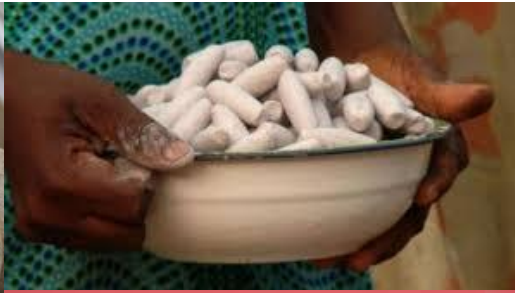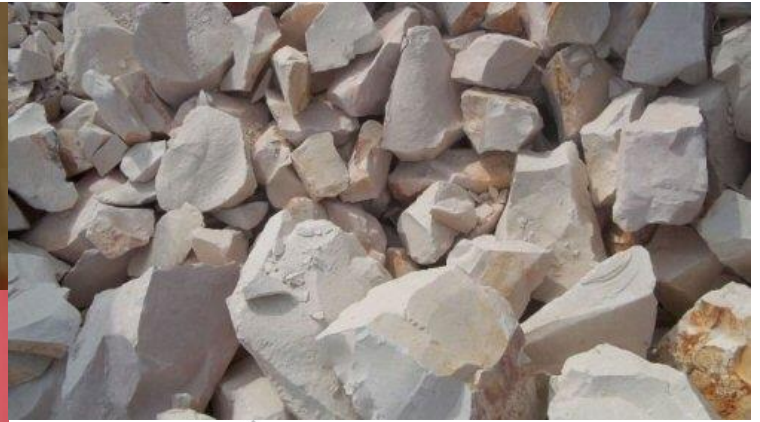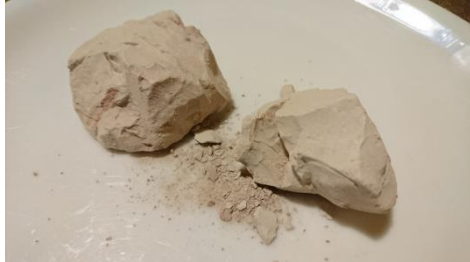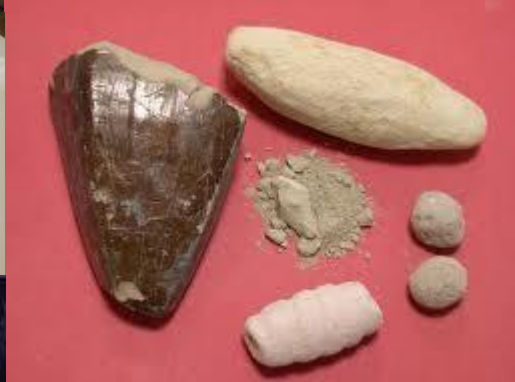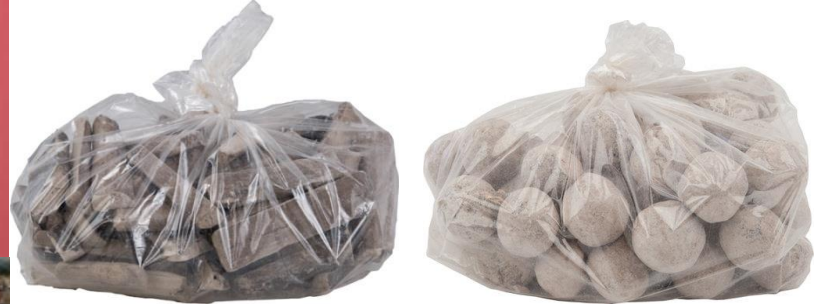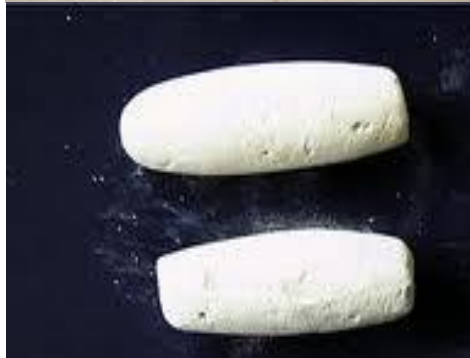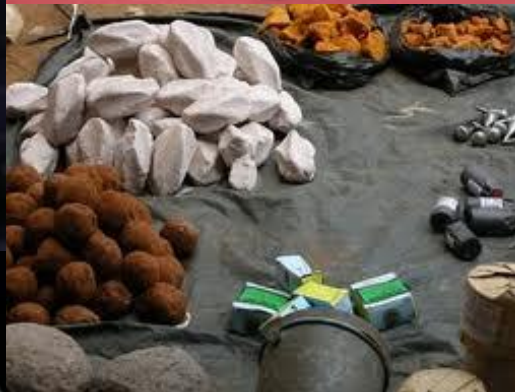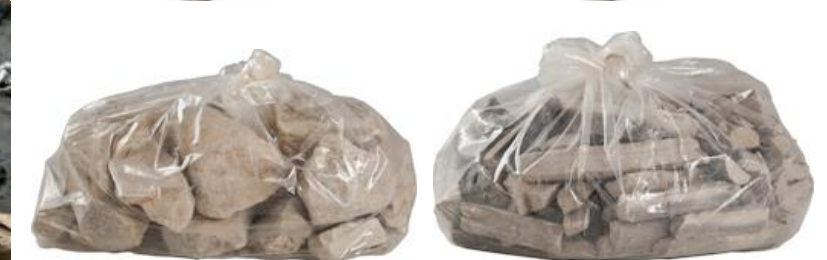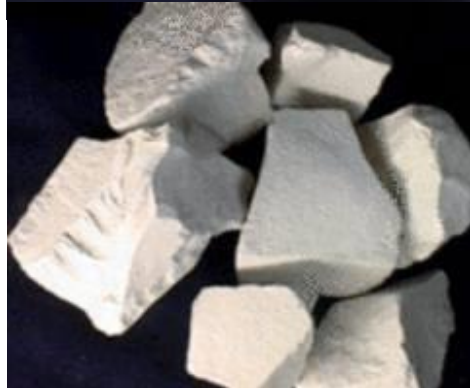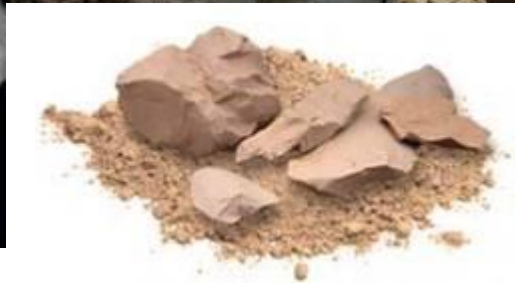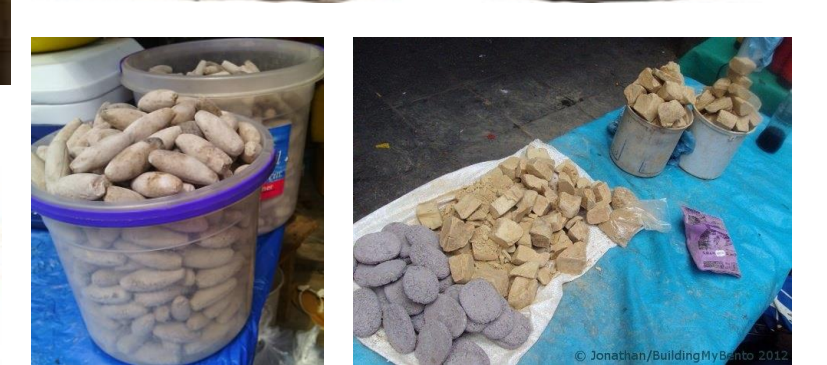

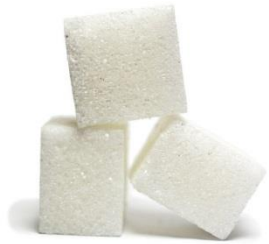

1 (<25g)

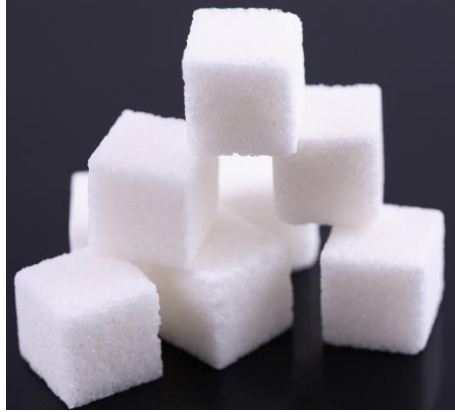

2 (25-<50g)

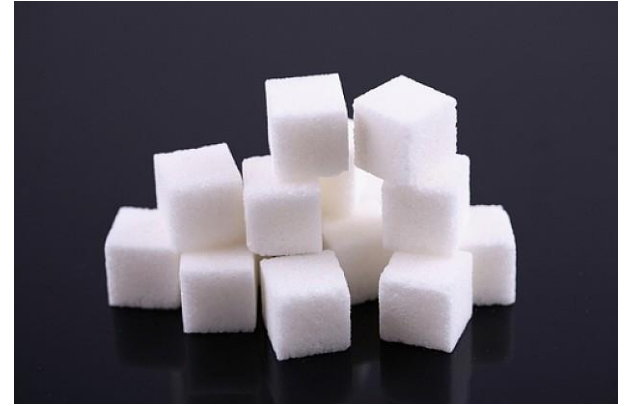

3 (50-<75g)

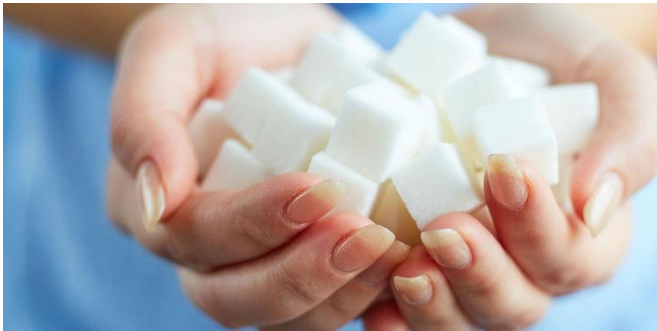

4 (75-<150g)

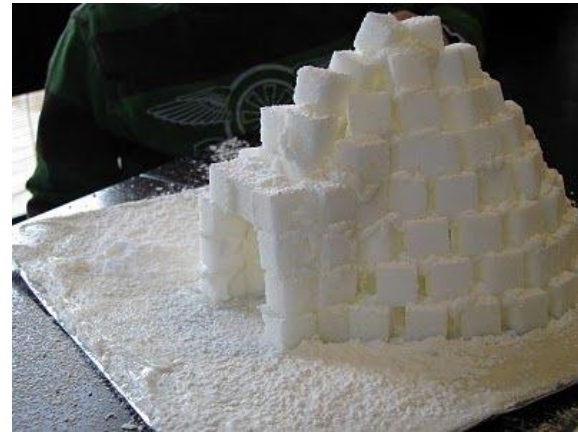

5 ( $\geq 150$ g)

Supplement: S3 Fig — This is the picture board used to identify kaolin consumption and evaluate quantities consumed. (PDF) [file pone.0220557.s003.pdf]

## Total within sum of square according to the number of clusters

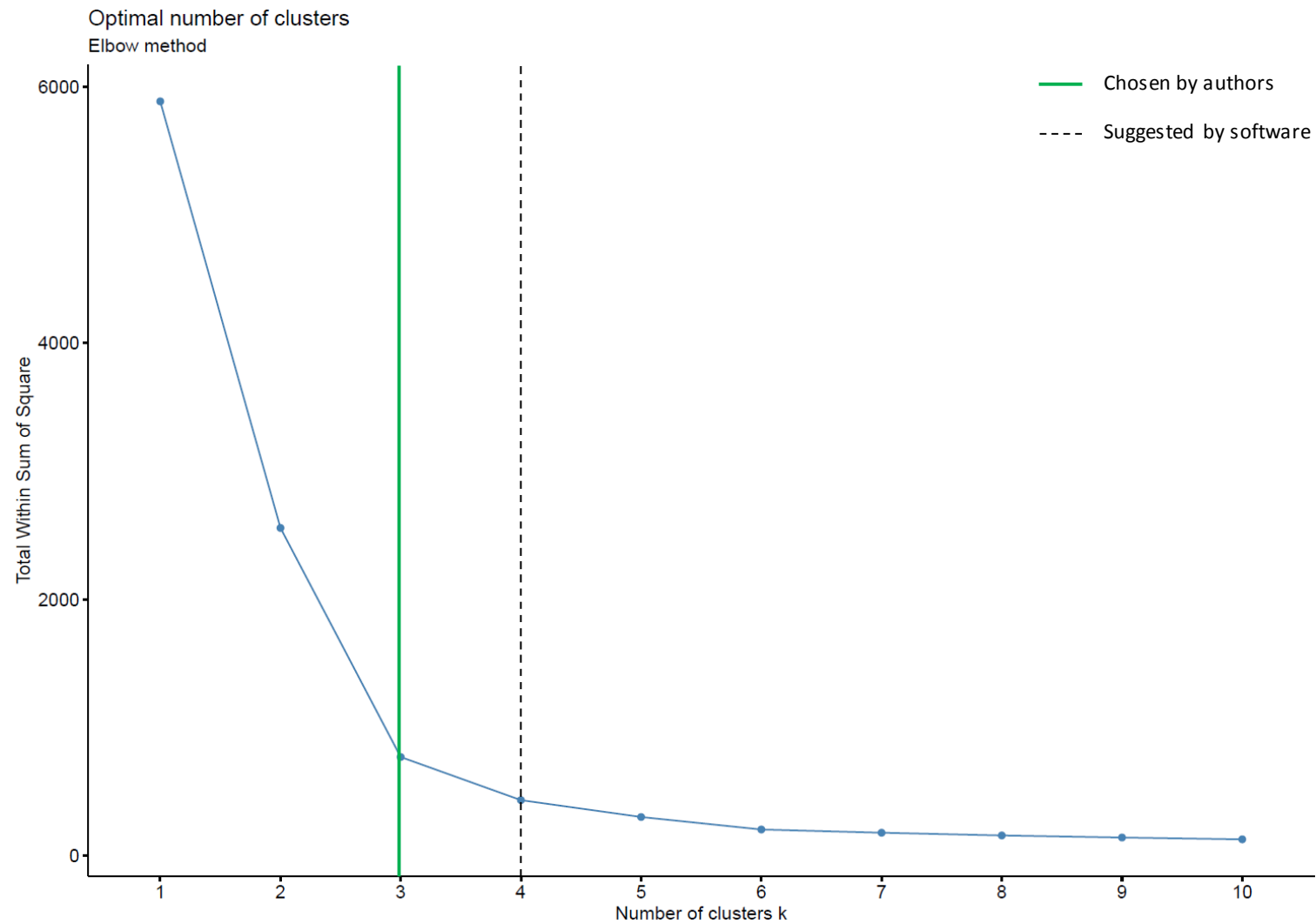

Supplement: S4 Fig — This figure shows how the 3-cluster solution was chosen. (PDF) [file pone.0220557.s004.pdf]
